# State Medical Society of Arkansas

**Published:** 1878-05-20

**Authors:** 


### STATE MEDICAL SOCIETY OF ARKANSAS.

The above society convened at Fort Smith, on May 1st., being its third annual session.

So much of the space of our present issue is given to our own State Association that we cannot give details of the State Medical Society of Arkansas. The attendance seems to have been reasonably good—with quite an accession of new members. There were interesting discussions, and a number of valuable papers reported. Several members from Hot Springs were expelled. The officers elected for the ensuing year, are:

For President—A. A. Horner, of Phillips county.

For Vice-Presidents—T. W. Hurley, of Benton county; W. H. Hawkins, of Little River county; J. S. Shibbley, of Logan county; Isarc Folsom, of Lonoke county.

For Secretary—R. G. Jennings.

For Assistant Secretary—L. P. Gibson.

For Librarian—J. H. Lenow.

Next place of meeting, Little Rock, the first Wednesday in May, 1879.

